# Hepatic epithelioid hemangioendothelioma, a rare liver tumor: a case report and review of the literature

**DOI:** 10.3389/fonc.2025.1522002

**Published:** 2025-05-16

**Authors:** Chao Deng, Yulong An, Jinli Liu, Chao Wang, Haoxian Gou, Jinpeng Zhang, Minghui Gu, Mengmeng Li, Tao Wang, Hao Luo

**Affiliations:** ^1^ Department of General Surgery, The General Hospital of Western Theater Command, Chengdu, China; ^2^ Clinical Medicine College of Southwest Medical University, Luzhou, Sichuan, China; ^3^ Department of Pathology, The General Hospital of Western Theater Command, Chengdu, China; ^4^ Department of Anesthesiology, The General Hospital of Western Theater Command, Chengdu, China

**Keywords:** hepatic epithelioid hemangioendothelioma, liver resection, microwave ablation, case report, review of the literature

## Abstract

**Introduction:**

Hepatic epithelioid hemangioendothelioma (HEHE) is classified as a rare, low-grade malignant neoplasm of vascular origin. This study presents a case report of a young female patient recently diagnosed with HEHE. A comprehensive review of the current understanding of HEHE is provided, along with the implementation of a novel combination treatment approach based on contemporary knowledge of this rare hepatic malignancy.

**Case:**

A 22-year-old female patient, with no prior medical history, underwent a routine health examination five months prior to presentation. Multiple space-occupying lesions in the liver were incidentally detected through ultrasound imaging, despite the patient being asymptomatic. Subsequent positron emission tomography-computed tomography (PET-CT) examination suggested the possibility of benign lesions. Recent follow-up examinations revealed no further progression of the lesions; however, a biopsy of the lesions confirmed the diagnosis of HEHE. Comprehensive imaging studies, including ultrasound, computed tomography (CT), and magnetic resonance imaging (MRI), demonstrated scattered space-occupying lesions throughout the liver, with no evidence of distant metastasis. To minimize surgical trauma and preserve liver function, a multidisciplinary team was consulted, and a treatment plan was devised: liver resection in combination with microwave ablation of the lesions. The patient’s postoperative recovery was uneventful, leading to successful hospital discharge.

**Conclusion:**

HEHE is characterized by its insidious onset and rarity, often presenting with multiple lesions at the time of clinical diagnosis. While liver transplantation may be considered the optimal treatment for multifocal HEHE, in cases where transplantation is not feasible, the combination of liver resection and microwave ablation of lesions may represent a safe and effective alternative therapeutic approach.

## 1 Introduction

HEHE is a rare neoplasm characterized by low to moderate malignancy, with an estimated incidence of approximately one case per million individuals (1). Due to its exceptionally low prevalence, well-defined diagnostic and treatment protocols for this condition have yet to be established. This article presents a case report detailing the diagnostic approach and therapeutic management of a patient diagnosed with HEHE, drawing upon previous clinical experiences.

## 2 Case presentation

The patient, a 22-year-old female of Han ethnicity, presented with a height of 158 cm and a weight of 45 kg. The unmarried patient, employed as a worker, reported a three-year history of tobacco use and a six-year history of alcohol consumption. No prior exposure to radioactive substances or carcinogens was reported, and the patient’s family history was negative for similar pathologies.

A hepatic mass was incidentally discovered during a routine examination five months prior to admission. Upon hospitalization, a comprehensive evaluation was conducted. The patient exhibited no subjective symptoms, and physical examination revealed no abnormalities. Except for mild abnormalities in alanine aminotransferase and aspartate aminotransferase in liver function tests, no abnormalities were found in other laboratory test indicators.

**Figure 1:**
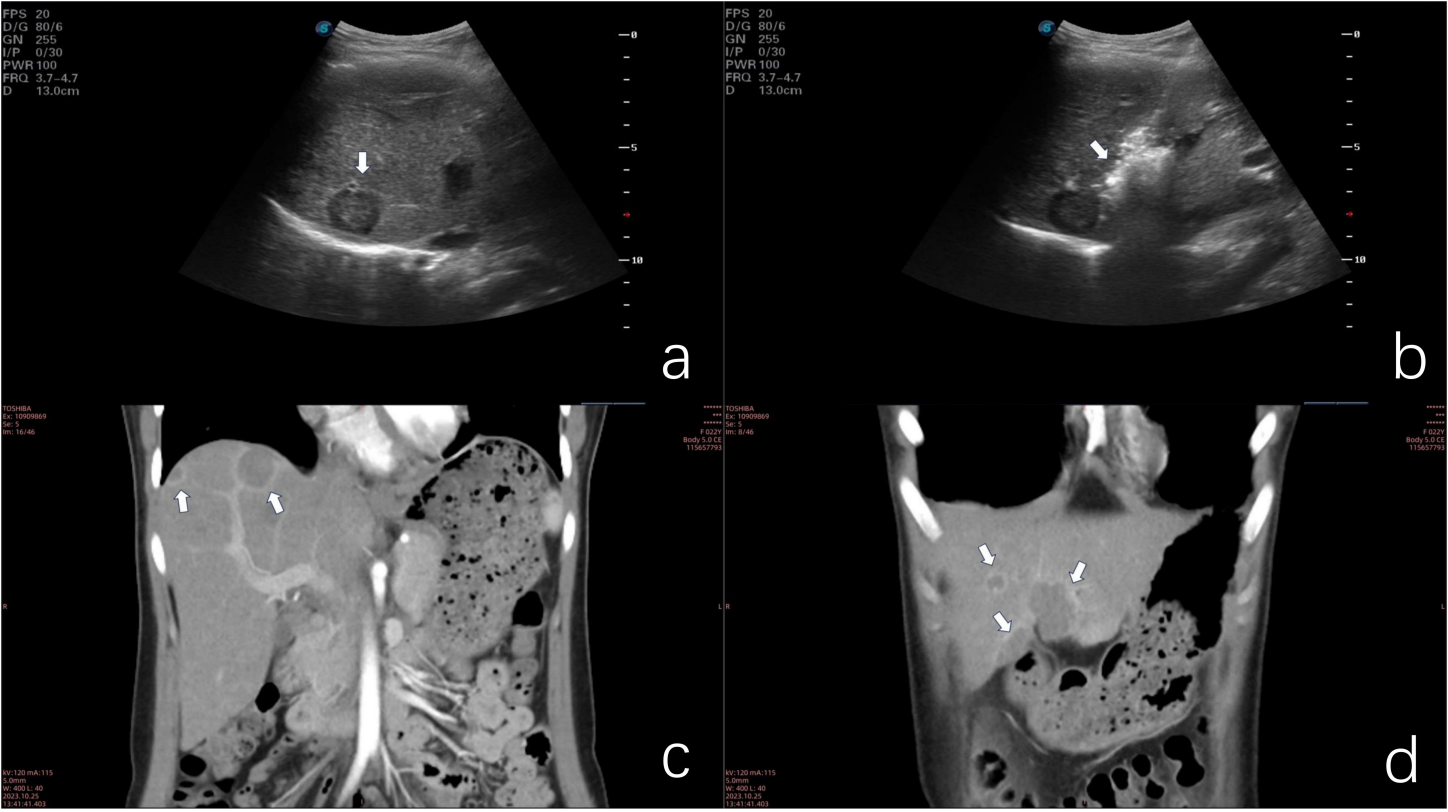
**(a)** Ultrasonographic image demonstrating hypoechoic nodular lesions. **(b)** Microwave ablation of a deep-seated lesion in the right anterior hepatic lobe. **(c)** Coronal contrast-enhanced CT scan illustrating the lollipop sign. **(d)** Coronal contrast-enhanced CT scan of confluent lesions in hepatic segments III and IV.

**Figure 2:**
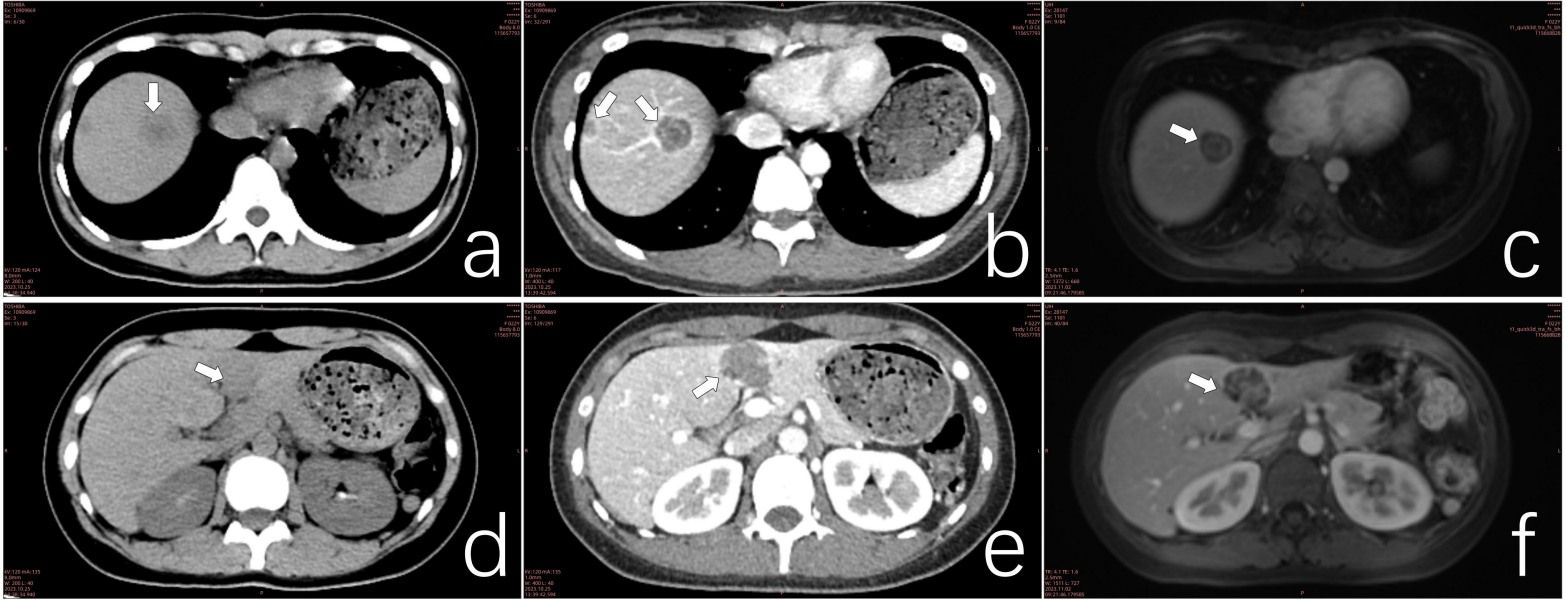
**(a)** CT scan of a lesion in hepatic segment VIII. **(b)** Contrast-enhanced CT scan of the segment VIII lesion exhibiting the target sign. **(c)** MRI of segment VIII lesions demonstrating the double ring sign. **(d)** CT scan of confluent lesions in hepatic segments III and IV. **(e)** Contrast-enhanced CT scan of confluent lesions in segments III and IV showing mild enhancement. **(f)** MRI of confluent lesions in segments III and IV, revealing heterogeneous internal signals.

Ultrasonographic examination revealed multiple hypoechoic lesions within the liver parenchyma. CT imaging demonstrated low-density lesions on unenhanced scans, with mild enhancement observed on contrast-enhanced studies. Several lesions exhibited distinctive target signs and lollipop signs. The neoplasms were distributed throughout both hepatic lobes. The largest lesion, a confluent mass located in the inferior aspect of liver segments III and IV, measured approximately 24.9*34.7*42.0mm (sagittal*axial*coronal planes). This lesion displayed patchy enhancement and heterogeneous density on imaging. MRI revealed ill-defined tumor margins with hypointense signals on T1-weighted sequences and hyperintense signals on T2-weighted sequences. The number of lesions identified on MRI correlated with the findings from contrast-enhanced CT scans (**Figures 1**, **2**).

The definitive diagnosis of HEHE was established through percutaneous liver biopsy.

**Figure 3:**
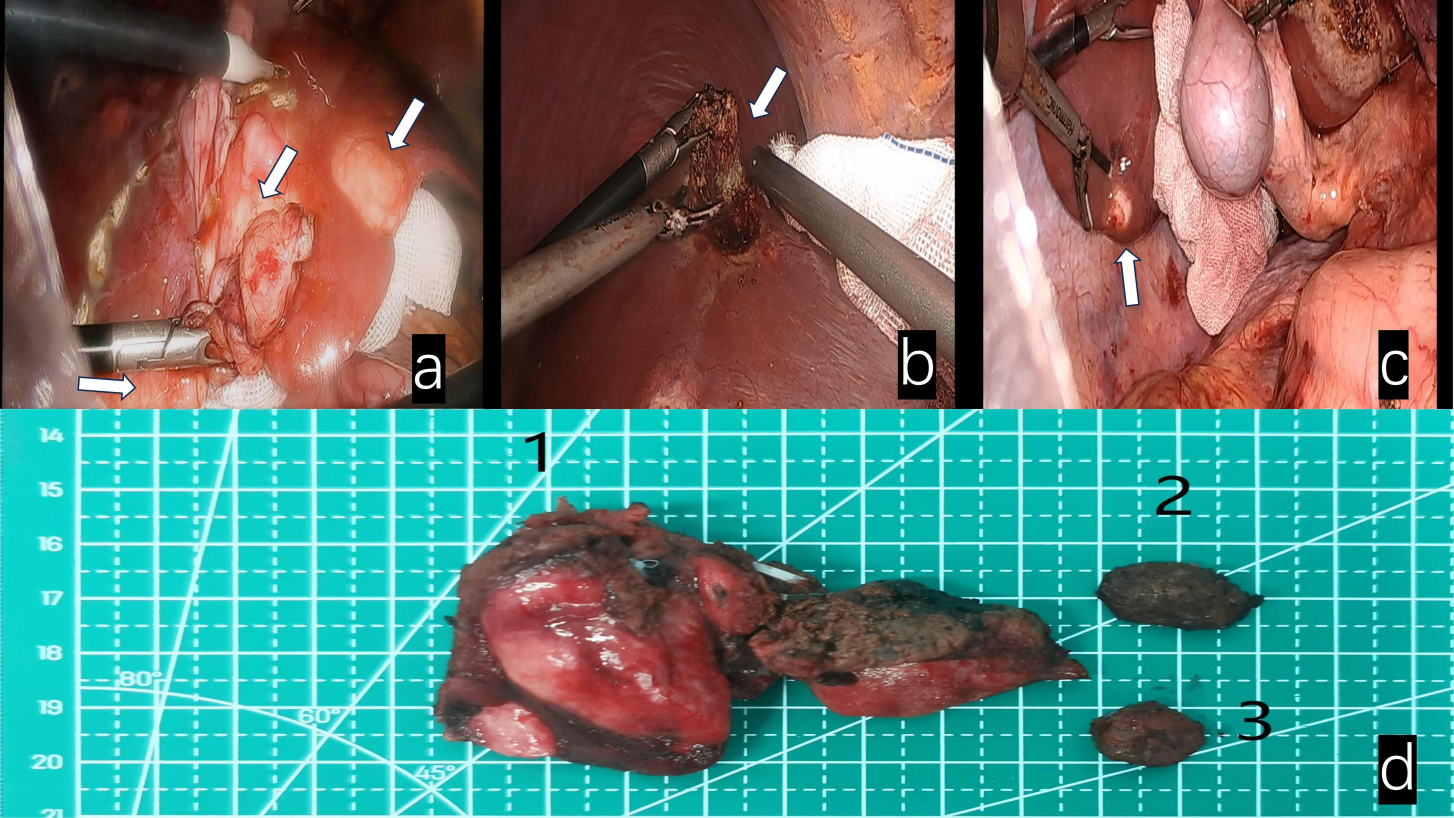
White arrows indicate: **(a)** Fusion lesions of liver segments III and IV. **(b)** Lesion in segment VIII of the liver. **(c)** Lesion in segment VI of the liver. **(d)** Surgical specimens 1. Fusion lesion specimens from liver segments III and IV; 2. Lesion specimen from liver segment VIII; 3. Lesion specimen from liver segment VI.

Given the rarity of this neoplasm and the absence of standardized treatment guidelines, a multidisciplinary team consultation was convened to formulate an optimal therapeutic strategy. The consensus treatment plan comprised microwave ablation for select lesions, coupled with surgical resection of the confluent masses in hepatic segments III and IV (**Figure 3**), as well as lesions in segments VI and VIII. Following a period of post-discharge convalescence, secondary ablation procedures were performed on the remaining lesions (**Figure 1b**).

Postoperative pathological examination revealed that specimens from the left liver, liver segment VI, and liver segment VIII were all confirmed as epithelioid hemangioendothelioma.

**Figure 4:**
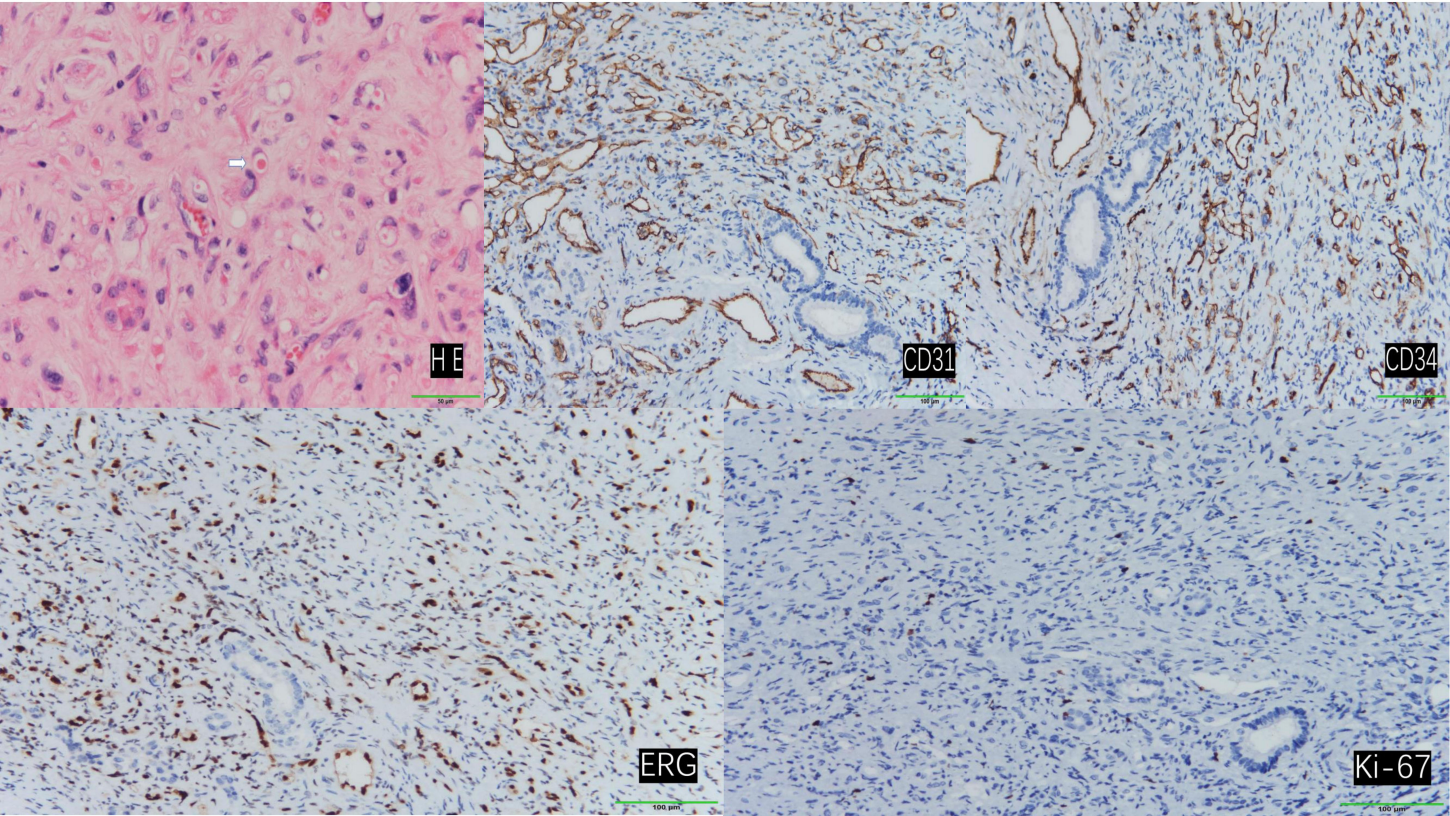
Hematoxylin and eosin (H&E) staining revealed tumor cells with abundant vacuolated cytoplasm and peripherally located nuclei, presenting a ring-like appearance. Occasionally, erythrocytes were observed within the vacuolated cytoplasm (indicated by arrows). (200×) Immunohistochemical staining demonstrated positive expression of CD31, CD34, ERG, and Ki-67. (100×).

Immunohistochemical analysis demonstrated that the tumor cells exhibited positive staining for CD31, CD34, and ERG. The Ki-67 proliferation index was determined to be 3%, and SMA displayed focal positivity. In contrast, CK, STAT-6, and Vim were found to be negative (**Figure 4**).

On the first day after surgery, the patient’s transaminase suddenly increased, which was considered to be caused by the destruction of liver cells by surgery and microwave therapy. Dynamic reexamination of transaminase within one week after surgery showed a stable downward trend. One month after discharge, the patient’s aminotransferase was basically back to normal. A CT scan one month after surgery showed that the ablation lesion was significantly smaller than before the surgery. The patient was followed up more than 16 months in another hospital for personal reasons, the disease has not progressed.

## 3 Discussion

HEHE is classified as a low-to-intermediate-grade malignancy, positioned between hemangioma and angiosarcoma in terms of aggressiveness (2). HEHE exhibits a higher prevalence in females, with an average age of onset around 40 years (3). The etiology of HEHE remains elusive; however, current research suggests associations with certain drugs, carcinogens, and biological factors (2, 4).

Clinical symptomatology and physical examination provide limited diagnostic value for HEHE. Lesions are typically detected incidentally through ultrasound or CT examinations (5–8). Ultrasonographic findings most commonly reveal hypoechoic masses, although heterogeneous echogenicity or hyperechogenicity may also be observed, depending on the tumor stage (5, 9). CT imaging of HEHE typically demonstrates multiple nodules or masses involving both hepatic lobes. Contrast-enhanced scans often reveal significant peripheral enhancement, with some lesions exhibiting characteristic features such as target sign, lollipop sign, and capsular retraction. The concurrent presence of these signs should raise suspicion for HEHE. As the disease progresses, lesions may coalesce, resulting in heterogeneous internal density on contrast-enhanced CT scans (5, 10–14). MRI findings in HEHE are comparable to those of CT; however, MRI offers superior detection of small lesions (15). PET-CT is valuable for evaluating extrahepatic lesions.

A definitive diagnosis of HEHE relies on histopathological examination. The tumor tissue comprises three cell types: epithelial cells, dendritic cells, and intermediate cells. Hematoxylin-eosin staining reveals tumor cells with vacuolated cytoplasm, occasionally containing erythrocytes. The nuclei are characteristically located at the cell periphery and exhibit intense staining (2, 16–18). Immunohistochemistry is essential for confirming the vascular origin of the tumor. Vascular markers CD31, CD34, and ERG typically demonstrate positive expression, while the Ki-67 index provides an initial assessment of tumor aggressiveness. Podoplanin staining aids in distinguishing HEHE from other hepatic malignancies. Keratin expression may be observed in some cases (1, 5, 18–25). Tumor markers, such as carbohydrate antigens, assist in differentiating primary HEHE from metastatic liver cancer (3, 26).

The t(1;3)(p36.3;q25) translocation, resulting in the CAMTA1-WWTR1 fusion product, represents the most prevalent genetic aberration in HEHE. The generation of YAP1-TFE3 fusion subsets has been associated with age of onset. Additionally, several clonal abnormalities have been identified in HEHE, including multiple complex translocations between chromosomes 7 and 22, a Robertsonian translocation of chromosome 14, and loss of the Y chromosome (19, 20, 27).

Among invasive treatment modalities, liver transplantation is widely recognized as the optimal therapeutic approach for hepatic malignancies. Studies focusing on HEHE have demonstrated superior survival rates for liver transplantation compared to non-transplantation treatment strategies (28, 29).

Liver resection is considered one of the optimal treatments, particularly for solitary localized HEHE (1). Radiofrequency and microwave ablation exhibit similar characteristics in the treatment of hepatic tumors, including ablation principles, safety profiles, and indications. However, radiofrequency ablation is generally limited to lesions smaller than 3 cm, while microwave ablation has virtually no such restriction. These treatment modalities have demonstrated comparable long-term survival and recurrence rates to liver resection (30, 31). The patient in this case presented with multiple, widely distributed lesions. Treating all lesions simultaneously would inevitably result in significant iatrogenic trauma and hepatic function impairment. A multidisciplinary consultation was conducted to address these challenges. To minimize damage and facilitate postoperative recovery, a decision was made to completely resect certain lesions and perform microwave ablation on larger lesions deep within the right hepatic lobe, reserving smaller lesions for subsequent ablation following patient recovery. The primary objective of the initial treatment was to maximize tumor reduction, while the secondary microwave ablation aimed to eliminate all visible lesions.

Pharmacological intervention may serve as an effective and tolerable treatment option for patients awaiting liver transplantation or those who have lost surgical candidacy. For instance, the combination of capecitabine and bevacizumab has been reported in the treatment of metastatic HEHE. Additionally, antitumor drugs such as sorafenib, or doxorubicin have been reported as alternative therapies in HEHE management, demonstrating varying therapeutic efficacies (26, 32).

HEHE is classified as a low-grade malignant neoplasm. Literature indicates a 5-year survival rate of 50% even in the absence of treatment (1). Unlike common hepatic malignancies, the presence of extrahepatic metastases in HEHE does not impact survival rates but is associated with treatment status (1, 33). Among treated patients, those who underwent surgical intervention demonstrated prolonged survival compared to those managed non-surgically (33). Factors potentially influencing prognosis include ethnicity, gender, age, Charlson-Deyo score, treatment status, tumor dimensions, intrahepatic metastasis, and transplantation waiting time (1, 18, 33–36). It is noteworthy that the majority of literature emphasizes the significance of surgical intervention.

## 4 Conclusion

HEHE is characterized by its insidious onset and rarity, often presenting with multiple lesions at the time of clinical diagnosis. While liver transplantation may be considered the optimal treatment for multifocal HEHE, in cases where transplantation is not feasible, the combination of liver resection and microwave ablation of lesions may represent a safe and effective alternative therapeutic approach.

## Data Availability

The datasets presented in this study can be found in online repositories. The names of the repository/repositories and accession number(s) can be found in the article/supplementary material.
